# Complications after hysterosalpingography with oil- or water-based contrast: results of a nationwide survey

**DOI:** 10.1093/hropen/hoz045

**Published:** 2020-01-15

**Authors:** Inez Roest, Nienke van Welie, Velja Mijatovic, Kim Dreyer, Marlies Bongers, Carolien Koks, Ben Willem Mol

**Affiliations:** 1 Department of Reproductive Medicine, Máxima Medical Centre, Veldhoven, Eindhoven, the Netherlands; 2 Department of Reproductive Medicine, Amsterdam University Medical Centre, Vrije Universiteit Amsterdam, Amsterdam, the Netherlands; 3 Grow Research School Oncology and Developmental Biology, Maastricht University, Maastricht, the Netherlands; 4 Department of Obstetrics and Gynaecology, University of Monash, Melbourne, Victoria, Australia

**Keywords:** hysterosalpingography, infertility, contrast, complications, intravasation, embolization, safety

## Abstract

**STUDY QUESTION:**

What is the incidence of complications after hysterosalpingography (HSG) using oil-based contrast versus water-based contrast?

**SUMMARY ANSWER:**

Among 5165 women undergoing HSG, the most frequently reported complication after HSG with oil- and water-based contrast was intravasation of contrast medium (4.8% versus 1.3%, respectively), which was without further consequences, and pulmonary embolization or death did not occur.

**WHAT IS KNOWN ALREADY:**

An HSG with oil-based contrast increases pregnancy rates in women with unexplained infertility. However, there have been some concerns regarding complications, including the risks of intravasation of the contrast medium, oil embolism and infection. Here, we present the incidence of complications after HSG with different types of contrast media used in the Netherlands in the year 2017.

**STUDY DESIGN, SIZE, DURATION:**

In January 2018, an electronic survey was sent to all 73 clinics in the Netherlands that perform HSG. The survey consisted of 12 questions addressing the number of HSGs performed in 2017, the amount and type of contrast medium used, the occurrence of post-procedural complications and what their clinical consequences were. Non-responding clinics were sent multiple reminders.

**PARTICIPANTS/MATERIALS, SETTING, METHODS:**

We calculated the incidence of the complications and reported on their clinical consequences. Furthermore, we examined the average amount of contrast used as well as the administration of prophylactic antibiotics.

**MAIN RESULTS AND THE ROLE OF CHANCE:**

The response rate was 96% (67/70) (during the study, one site closed and was not included while two clinics no longer performed HSGs). In the 67 clinics, 3289 HSGs with oil-based contrast and 1876 HSGs with water-based contrast were performed in 2017. The median amount of contrast used was 8.0 ml (interquartile range (IQR) 7.0–10.0) for oil-based contrast and 10.0 ml for water-based contrast (IQR 10.0–10.0). Antibiotic prophylaxis was administered in 61% (41/67) of the clinics. Intravasation occurred in 4.8% of the HSGs performed with oil-based contrast and in 1.3% of the HSGs with water-based contrast (relative risk (RR), 3.6; CI, 2.4–5.4). Pulmonary embolism or death was not reported. Pelvic inflammatory disease (PID) occurred in 0.3% of the HSGs performed with oil-based contrast versus 0.4% with water-based contrast. PID occurred in 0.3% of the HSGs in clinics using antibiotic prophylaxis and 0.2% in clinics not using antibiotic prophylaxis. Allergic reactions were reported in one HSG performed with oil-based contrast (0.03%) compared with two HSGs performed with water-based contrast (0.1%). Anaphylactic reactions did not occur. The overall complication rate was 5.1% in the clinics that used oil-based contrast versus 1.8% in the clinics that used water-based contrast (RR, 2.8; CI, 1.9–4.0; *P*-value, <0.0001).

**LIMITATIONS, REASONS FOR CAUTION:**

Half of the clinics did not routinely register complications, and the incidence of the complications in their clinic was based on the recall of the clinician. Estimated complication rates in the clinics with and without systematic registration did not significantly differ. The survey asked about the frequency of intravasation but no classification system is being used in daily practice, which may create differences in reporting. There was no standard screening of post-HSG thyroid function for the mother and the foetus.

**WIDER IMPLICATIONS OF THE FINDINGS:**

In this nationwide cohort study, the complication rates after HSG were low. Intravasation occurred more frequently with the use of oil-based contrast compared with water-based contrast but did not lead to any problems or symptoms in any of the women. We therefore conclude that safety concerns should not be a reason to deny the use of oil-based contrast in women with unexplained infertility. The data also support that fluoroscopy appears to be an essential safety measure during HSG.

**STUDY FUNDING/COMPETING INTEREST(S):**

This work was partly funded by Guerbet, France. I.R. reports receiving travel fee for presenting at the Congress of the American Society for Reproductive Medicine 2019 from Guerbet. V.M. reports receiving travel and speaker’s fee as well as research grants from Guerbet. K.D. reports receiving travel and speaker’s fee from Guerbet. B.W.M. is supported by an National Health and Medical Research Council (NHMRC) Practitioner Fellowship (GNT1082548). B.W.M. reports consultancy for ObsEva, Merck KGaA and Guerbet and travel and research grants from Merck KGaA and Guerbet. The other authors do not report conflicts of interest.

**TRIAL REGISTRATION NUMBER:**

N19.056.

**WHAT DOES THIS MEAN FOR PATIENTS?**

Hysterosalpingography (HSG) is a widely used medical test that checks whether the tubes (fallopian tubes) that join the ovary to the womb are open. This is done as an outpatient procedure in women who are having difficulty becoming pregnant (infertile women). During this procedure, a liquid is flushed through the womb and fallopian tubes and a type of photograph is taken (radiograph). The liquid used is called contrast media and it is dissolved either in water (water-based) or in oil (oil-based). This research group recently found that in infertile women undergoing this procedure, flushing with oil-based contrast media liquid results in more pregnancies and live births than flushing with water-based contrast media liquid.

In this study, we looked at the safety of these two types of contrast media by measuring the complications reported in women after HSG procedures with the different types of contrast in 2017 in the Netherlands. The most frequently reported complication was finding contrast material in the vessels around the uterus, which is called intravasation. This happened in 4.8% of the HSGs with oil-based contrast and in 1.3% of the HSGs with water-based contrast. Since this complication does not lead to any problems or symptoms in any of the women, we conclude that both types of procedure are considered to be safe.

## Introduction

Knowledge of tubal patency during the fertility workup is essential for the choice of treatment. Hysterosalpingography (HSG) is the most commonly used diagnostic method to test tubal patency in patients suffering from infertility (National Institute Care Excellence (NICE), 2017). Over the years, both water- and oil-soluble contrast media have been used. In 2017, a large randomized controlled trial (RCT) showed that an HSG with the use of oil-based contrast medium (Lipiodol® Ultra-Fluid) results in a 10% higher ongoing pregnancy rate, within 6 months after the procedure, compared with an HSG with water-based contrast (39.7% versus 29.1%; RR, 1.4; 95% CI, 1.2–1.6; *P* < 0.001) (Dreyer *et al.*, 2017). Afterwards, two systematic reviews with meta-analyses confirmed these favourable effects of oil-based contrast media on pregnancy and live birth rates (Fang *et al.*, 2018; Wang *et al.*, 2019).

Given these favourable results on fertility, HSG with oil-based contrast is preferred. However, there is a concern about the risk of complications from using oil-based contrast media during HSG. The most frequently mentioned concerns are the possible risks of venous intravasation and, as a result of that, embolism, the risk of a pelvic infection and maternal/foetal risks of thyroid dysfunction (Uzun *et al.*, 2004; Kaneshige *et al.*, 2015; Satoh *et al.*, 2015; So *et al.*, 2017).

It is known that the risk of intravasation is higher with the use of oil-based contrast as compared with water-based contrast. A recent meta-analysis reporting on 793 women found an odds ratio (OR) of 5.1 (95% CI, 2.3–11.2) for the risk of intravasation with the use of oil-based contrast compared with water-based contrast, with all intravasations being clinically asymptomatic (Wang *et al.*, 2019). Another meta-analysis of 1179 HSGs performed with oil-based contrast similarly reported no cases of embolism, granulomas or allergic reactions (Fang *et al.*, 2018). Since sample sizes of these meta-analyses were relatively low, concern regarding complications of oil-based contrast media remains. Therefore, we evaluated the incidence of complications after HSG and their possible consequences through a nationwide survey.

## Materials and Methods

An electronic survey was sent to all 73 clinics in the Netherlands that perform HSG, addressed to gynaecologists specialized in reproductive medicine. The clinics that did not respond on the first attempt were approached again by telephone or e-mail.

The questionnaire (Supplementary Data) consisted of 12 questions regarding the number of HSGs performed in 2017, the type and average amount of contrast medium used and the occurrence of post-procedural complications as well as their clinical consequences. Fluoroscopy screening is routinely used during HSGs in the Netherlands. Questions were asked on the frequencies of the following complications: allergic reactions, anaphylactic reactions, intravasation of the contrast medium, embolisms and pelvic inflammatory disease (PID) as well as other complications that had occurred post-HSG. No description of the terms was included in the survey. Furthermore, information on the standard registration of complications, the use of antibiotic prophylaxis and standard safety precautions was requested. If a clinic had no standardized complication registry, the respondent was asked to provide an estimated number of complications based on their recall. The study was approved by the Institutional Review Board of the Máxima MC (reference number: N19.056).

**Table I:** **The incidence of the reported complications for HSGs with oil- and water-based contrast, as reported in a nationwide survey of clinics**.

	Oil-based contrast (N = 3289)	Water-based contrast (N = 1876)	Relative risk (95% CI)	p-value^*^
Intravasations	157 (4.8%)	25 (1.3%)	3.6 (2.4–5.4)	<0.0001
Embolisations	0 (0%)	0 (0%)	-	-
Allergic reactions	1 (0.03%)	2 (0.1%)	0.3 (0.03–3.1)	0.30
Anaphylactic reactions	0 (0%)	0 (0%)	-	-
Pelvic inflammatory disease	9 (0.3%)	7 (0.4%)	0.7 (0.3–2.0)	0.54
Sum of complications	167 (5.1%)	34 (1.8%)	2.8 (1.9–4.0)	<0.0001
Women without any complication	3122 (94.9%)	1842 (98.2%)	1.0 (0.96–0.98)	<0.0001

^*^Pearson chi-square test or Fisher’s exact test as appropriate.

Categorical data were reported as absolute numbers and percentages. RRs and 95% CI were calculated for binary outcome measurements. The Pearson chi-square test or Fisher’s exact test were used as appropriate. For normally distributed continuous variables, means with SDs were summarized; non-normally distributed continuous variables were represented as medians with interquartile ranges (IQRs). Continuous outcomes were analysed with the use of an independent *t*-test or the Mann–Whitney U-test, as appropriate. A *P*-value of <0.05 was considered to indicate statistical significance. The data were analysed by IBM SPSS Statistics, version 24 (IBM-corporation, Armonk, NY, USA).

## Results

From January 2018, 73 questionnaires were sent out. During the study, one site was closed and was not further included in the study while two clinics responded that they did not perform HSGs. The response rate was 96% (67/70 clinics). In the 67 clinics, a total of 5165 HSGs had been performed in 2017. The number of HSGs per clinic varied between 3 and 328.

In 44 clinics, an oil-based contrast (Lipiodol® Ultra-Fluid) was used; of which 29 clinics used Lipiodol® Ultra-Fluid for the whole year, while 15 clinics had started using Lipiodol® Ultra-Fluid since mid-2017. The total amount of HSGs performed with Lipiodol® Ultra-Fluid in 2017 was 3289. The years of experience with Lipiodol® Ultra-Fluid ranged from 0.5 year to more than 20 years, with a median of 3 years (IQR 0.5–10.0).

Twenty-one clinics responded that they only used water-based contrast during HSG. The following water-based contrast media were used: Omnipaque® (six clinics), Ultravist® (four clinics), Visipaque® (three clinics), Iomeron® (two clinics), Hexabrix®, Telebrix®, Xenetix®, Omnipaque®/Visipaque® (one clinic), while the remaining two clinics did not specify the type of water-based contrast media used. The total amount of HSGs performed with water-based contrast medium in 2017 was 1876.

The remaining two clinics responded that they used a combination of the two contrast media: they perform an extra flushing with Lipiodol® Ultra-Fluid after diagnosing tubal patency with the use of water-based contrast.

The median amount of contrast used was 8.0 ml (IQR 7.0–10.0) in the clinics that used oil-based contrast and 10.0 ml (IQR 10.0–10.0 ml) in the clinics that used water-based contrast (*P*-value, 0.09). One clinic used an infusion pump, while the other 66 clinics performed a manual instillation of the contrast medium.

In 54% of the clinics (36/67), complications were registered in the electronic patient file. A total of 37% of the clinics (25/67) did not register the complications that occurred during HSGs. Six clinics did not respond to this question.

The reported complications that occurred after HSGs with oil- and water-based contrast are displayed in Table I. The overall complication rate was 5.1% in the clinics that used oil-based contrast versus 1.8% in the clinics that used water-based contrast (RR, 2.8; CI, 1.9–4.0; *P*-value, <0.0001). The most reported complication was intravasation, which occurred in 4.8% of the HSGs performed with oil-based contrast and 1.3% of the HSGs performed with water-based contrast (RR, 3.6; CI, 2.4–5.2; *P*-value, <0.0001). All cases with intravasation were reported as asymptomatic, without leading to pulmonary embolism or death. In 0.3% of the HSGs with oil-based contrast and in 0.4% of the HSGs with water-based contrast, a PID occurred (RR, 0.7; CI, 0.3–2.0; *P*-value, 0.54). In these cases, antibiotic treatment and/or hospital admission took place. Anaphylactic reactions were not reported at all. Allergic reactions occurred in one of the HSGs with the use of oil-based contrast and two with water-based contrast (RR, 0.3; CI, 0.03–3.1; *P*-value, 0.30). One clinic reported vasovagal reactions as other complications (8.7%, 15/172 women).

The complication rate was not significantly different between clinics with and without a standard registration of complications. The incidence of intravasation in 36 clinics with standard registration was 4.4% (62/1412) with oil-based contrast and 1.3% (15/1131) with water-based contrast, compared with 5.6% (88/1579) and 1.3% (10/745) in 25 clinics without standard registration, respectively. The RR of intravasation when comparing clinics with standard registration to clinics without registration was 0.8 (CI, 0.6–1.1; *P*-value, 0.14) with oil-based contrast and 1.0 (CI, 0.5–2.2; *P*-value, 1.00) with water-based contrast.

### Antibiotic prophylaxis

In 61% of the clinics (41/67) antibiotic prophylaxis was prescribed, while 39% of the clinics (26/67) did not use prophylactic antibiotics. Among the clinics that prescribe antibiotics, there were different indications for prophylactic antibiotics.

In 15% of the clinics (6/41 444 women), all patients received antibiotic prophylaxis (risk of infection 0.0% (0/444)), while in 61% of the clinics (25/41 2533 women), patients with a high risk of tubal pathology (based on their medical history, a positive chlamydia antibody titre (CAT) or a positive sexual transmitted disease (STD) Polymerase chain reaction (PCR)) received antibiotic prophylaxis (risk of infection 0.3% (6/2389)). In 12% of the clinics (5/41 308 women) also patients with an unknown STD PCR outcome or unknown CAT received antibiotic prophylaxis (risk of infection 0.6% (2/308)). In 7% of the clinics (3/41, 259 women), antibiotics were prescribed after the procedure, in case of tubal pathology diagnosed on HSG (risk of infection 0.4% (1/259)). Two clinics (2/41) did not specify the indication for antibiotic prophylaxis, and in these clinics, the risk of infection was 1.8% (3/171). The overall incidence of PID in the clinics who used prophylactic antibiotics was 0.3% (12/3571). The incidence of PID in the clinics that did not routinely prescribe antibiotic prophylaxis was 0.2% (4/1949).

### Safety precautions

In 40% of the clinics (27/67), a crash cart was available in the HSG room to provide first aid in case of, for example, an allergic reaction, while 55% (37/67) did not have such a facility available during the performance of HSGs. Three clinics did not respond to this question.

## Discussion

In this nationwide retrospective analysis of 5165 women undergoing HSG, the overall complication rate was 5.1% after HSG was performed with oil-based contrast and 1.8% with water-based contrast. The most frequently reported complication was intravasation in 4.8% of the HSGs performed with oil-based contrast and 1.3% of the HSGs performed with water-based contrast (OR, 3.6; 95% CI, 2.4–5.4; *P*-value, <0.0001). All cases of intravasation were asymptomatic. In this analysis of 5165 women undergoing HSGs, oil embolisms or other clinical consequences of intravasation were not observed.

### Limitations

The main limitation of our study is its retrospective design. Because half of the clinics have no standard complication registration, the incidence of the HSG-related complications presented here is partly based on the recall of the clinician. However, the estimated rates in the clinics with and without systematic registration did not significantly differ. The incidence of thyroid dysfunction after HSG is not reported because no standard screening of post-HSG thyroid function is performed in the Netherlands. The survey asked about the frequency of intravasation; however, no classification system is being used in daily practice, which may create differences in reporting. Intravasation is defined as the passage of contrast media into the veins or the lymphatics, and an intravasation severity score was proposed in 2013 (Dusak *et al.*, 2013); however, this system has not been implemented in daily practice.

### Clinical implications

A higher incidence of intravasation in HSGs with oil-based contrast media in comparison with water-based contrast media, as shown in our results, is in line with recent evidence. A recent meta-analysis of three RCTs, reporting on 793 HSGs, compared the risk of intravasation with oil-based contrast to the risk with water-based contrast. Pooling the data of these three studies showed that, compared with water-based contrast, oil-based contrast was associated with higher odds of intravasation (OR, 5.1; 95% CI, 2.3–11.2). All reported intravasations in this meta-analysis were asymptomatic without clinical consequences of an oil embolism (Wang *et al.*, 2019).

Despite this reassuring evidence, case reports are published on serious consequences of intravasation, including a pulmonary and cerebral oil embolus after an HSG with oil-based contrast: a remarkable fact is that no fluoroscopy guidance was used during this HSG (Uzun *et al.*, 2004). This is of importance as real time fluoroscopy guidance might prevent oil embolism and its clinical consequences, when discontinuation of the HSG procedure is accomplished, in case of intravasation. This is confirmed in our study, which evaluates 5165 HSGs performed with fluoroscopy and shows no symptomatic intravasations. Therefore, fluoroscopy appears to be an essential safety measure during HSG.

Older publications suggest an increased incidence of symptomatic intravasation if large volumes of contrast medium are used. An amount of 4–6 ml with a maximum pressure of 180–200 mmHg was suggested (Eisen and Goldstein, 1945). Our study shows that the median amount of contrast used is 8.0 ml for the oil-based contrast (IQR, 7.0–10.0) and 10.0 ml for the water-based contrast (IQR, 10.0–10.0), compared with 9.0 ml (IQR 5.7–15.0) and 8.0 ml (IQR 5.9–13.0) for oil- and water-based contrast, respectively, in the H2Oil study (Dreyer *et al.*, 2017). Hypothetically, a higher amount of contrast could increase the risk of intravasation due to elevated pressure in the uterus. However, both in our study and the H2Oil study, embolization was not reported.

In none of the more than 5000 HSGs performed did an anaphylactic reaction occur. The incidence of PID was low and did not differ between clinics that routinely prescribe antibiotic prophylaxis or not and was also not different after HSGs with oil-based or water-based contrast. In the literature, a PID incidence of 0.5% after an HSG with antibiotic prophylaxis and 1.4% without prophylaxis is described (Li *et al.*, 2018). In addition, two RCTs (Rasmussen *et al.*, 1991; Lindequist *et al.*, 1994) reported on PID after HSG using water-based compared with oil-based contrast. Pooled analysis showed that there was insufficient evidence of differences in PID incidence between these two contrast media (OR, 0.23; 95% CI, 0.04–1.27) (Wang *et al.*, 2019).

Both types of contrast contain iodine, which can affect thyroid function after HSG (Mekaru *et al.*, 2008). The concentration of iodine is higher in oil-based contrast (480 mg iodine/ml) as compared with water-based contrasts (240–300 mg iodine/ml, depending on the pharmaceutical company). A cohort study demonstrated that women who had a subclinical hypothyroidism prior to HSG have a higher risk of developing hypothyroidism after an HSG, compared with euthyroid women, 35.7% versus 2.2%, respectively (Mekaru *et al.*, 2008). Subclinical hypothyroidism has been associated with different pregnancy complications, including an increased risk of (recurrent) miscarriage (van den Boogaard *et al.*, 2011). However, two meta-analyses comparing oil-based with water-based contrast did not show an increased risk of miscarriage with the use of oil-based contrast (Fang *et al.*, 2018; Wang *et al.*, 2019).

Fifteen of the 44 clinics that use oil-based contrast started using oil-based contrast in mid-2017, which is shortly after the publication of the H2Oil study results (Dreyer *et al.*, 2017). We can conclude that this publication has significantly changed the daily practice of fertility clinics in the Netherlands.

Despite the fact that there were no consequences of intravasation in our study, we advise to use the minimum amount of contrast needed for the diagnosis of tubal patency. Furthermore, we suggest additional research in the form of prospective studies on thyroid function after HSG in a western population and studies on the mechanism of action of oil-based contrast (Lipiodol® Ultra-Fluid).

## Conclusion

This retrospective analysis of 5165 HSGs performed in the Netherlands during a single book year, 2017, shows that serious complications after HSGs using oil-based contrast media and water-based contrast media are rare. The most frequent complication was intravasation (4.8% with oil-based contrast, 1.3% with water-based contrast), without any clinical consequences. Presumably, the use of fluoroscopy during HSGs may contribute to the prevention of embolism and death due to intravasation. The incidence of maternal and neonatal thyroid dysfunction is not reported. Further research, especially on maternal and neonatal thyroid function after HSG in a western population, is needed.

## Supplementary Material

Supplementary_Data_final_hoz045
