# Supplementary material for: Complications after hysterosalpingography with oil- or water-based contrast: results of a nationwide survey
Source: Hum Reprod Open. 2020 Jan 15;2020(1):hoz045. doi: 10.1093/hropen/hoz045 (PMC6964222; doi:10.1093/hropen/hoz045)
Supplement: Supplementary_Data_final_hoz045 [file supplementary_data_final_hoz045.docx]

**Supplementary Data**

The nationwide survey used to investigate complications after hysterosalpingography with oil or water-based contrast.

1. What is your position?

Gynaecologist

Gynaecologist in training (in Dutch: AIOS)

Doctor not in training for gynaecologist (in Dutch: ANIOS)

Fertility doctor

Other:

1. How many HSGs did your clinic perform in 2017? HSG’s
2. Who described the HSG images?
    Gynaecologist

Radiologist

Gynaecologist and radiologist

Other:

1. Does your clinic use an infusion pump for the HSG procedure?
    Yes

No, the contrast is infused manually

1. Which contrast medium does your clinic use standardly during HSGs?

Oil-based contrast

Water-based contrast, namely: _________________

1. How long has your clinic been using oil-based contrast during HSGs?

Since months/years

1. What is the average amount of contrast used per HSG procedure _____ mL.
   (1 ampoule Lipiodol contains 10 mL)
2. Which maternal complications have occurred after HSGs? And how often did they occur?

| Complication: | Frequency in 2017:  *In case no exact number of complications of 2017 is known, make an estimation of the* frequency. |
| --- | --- |
| Allergic reactions | times/year |
| Anaphylactic shock | times/year |
| Intravasation | times/year |
| Embolisation | times/year |
| Pelvic Inflammatory Disease (PID) | times/year |
| Thyroid dysfunction | times/year |
| Other: | times/year |
| No complications | times/year |

1. Is there a standardized registration of the maternal complications?
   Yes, they are registered on:
    No
2. What were the consequences of these complication(s)?
   Antibiotic treatment (per os / intravenous)
   Follow-up as an outpatient
   Hospital admission, amount of days:
3. Do you provide antibiotic prophylaxis before or after the HSG, and for which indications?
    Yes, indications:
    No
4. Is a crash cart available in the room where the HSGs are performed?

Yes, this crash cart contains:
 No

Thank you for your cooperation!

For questions and remarks mail to: Inez.roest@mmc.nl
